# Association of maternal characteristics with child feeding indicators and nutritional status of children under-two years in Rural Ghana

**DOI:** 10.1186/s12887-022-03651-1

**Published:** 2022-10-07

**Authors:** Christiana Nsiah-Asamoah, George Adjei, Samuel Agblorti, David Teye Doku

**Affiliations:** 1 Department of Clinical Nutrition and Dietetics, University of Cape Coast, Cape Coast, Ghana; 2 Department of Community Medicine, University of Cape Coast, Cape Coast, Ghana; 3 Department of Population and Health, University of Cape Coast, Cape Coast, Ghana

**Keywords:** Maternal factors, Under-two’s, Child feeding indicators, Nutritional status, Rural districts

## Abstract

**Background:**

Optimal nutrition during the first two years of a child’s life is critical for the reduction of morbidity and mortality. In Ghana, majority of children miss out on optimal nutrition and only few (13%) of children receive a Minimum Acceptable Diet (MAD). Several studies have investigated the influence of community-level factors on infants and young children feeding (IYCF) practices. However, little is known about the influence of maternal factors on IYCF practices in rural settings. Therefore, this study assessed the influence of maternal factors on the feeding indicators and nutritional status of children aged 6–23 months in two administrative districts in Ghana.

**Methods:**

Data were collected among 935 mothers who had children aged 6–23 months and accessed 21 Child Welfare Clinics within the study area. The study involved a face- to-face interview using structured questionnaires to capture maternal characteristics, dietary intake and anthropometric measurements of children. Multivariate logistic regression was used to study the association between maternal factors and child nutrition outcomes (MAD, dietary diversity score (DDS) and anthropometric indicators) using Stata 16.0 software.

**Results:**

Being employed (AOR = 3.07, 95% CI: 1.71—5.49, *p* < 0.001) and attaining secondary or higher education (AOR = 2.86, 95% CI: 1.42—5.78, *p* = 0.003) were significant predictors of children receiving MAD. Similarly, having an average decision-making autonomy increased the child’s odds of receiving MAD (AOR = 1.68, 95% CI: 1.02—2.76, *p* = 0.040). Children of mothers who attained secondary or a higher level of education (AOR = 0.59, 95% CI: 0.36 -0.97, *p* = 0.040) and those whose mothers were employed (AOR = 0.71, 95% CI: 0.47—1.07, *p* = 0.043) were associated with a reduced risk of underweight and stunting respectively. Children of mothers with average financial independence status were more likely to receive diversified meals (AOR = 1.55, 95% CI: 1.01–2.38, *p* = 0.045).

**Conclusions:**

High educational level and being employed have positive influence on MAD, stunting and underweight of children. High decision-making power and average financial independence of mothers are good predictors of children receiving MAD. Family planning, women empowerment in decision-making, providing employment opportunities for mothers and promoting girl-child education are recommended.

## Background

The first months of a child’s life (0–23 months) are a very critical phase during which rapid physical and mental development occurs [1]. Consequently, the United Nations Children’s Fund (UNICEF) advocates for and recommends age-appropriate complementary foods and feeding practices to support both physical growth and brain development during the early days of life, before a child attains two years [2].

According to the 2015 Ghana Demographic and Health Survey (GDHS), 19% of children under five years of age were stunted (short for their age), 5% were wasted (thin for their height), and 11% were underweight (low weight for their age) [3]. The national prevalence rates for stunting, wasting and underweight indicate that some positive strides have been achieved in Ghana, particularly when these rates are benchmarked against those in other African countries. However, these figures are still higher than the World Health Organization’s (WHO’s) classification of low prevalence [4]. Again, the prevalence figures for stunting and wasting are higher than the thresholds developed by the WHO–UNICEF Technical Expert Advisory Group on Nutrition Monitoring [5]. Therefore, notwithstanding the progress made, there is still the need to investigate the drivers of child malnutrition in Ghana. This has become necessary in view of the fact that there has been no steady downward trend of the indicators, particularly those that assess compliance to recommended Infant and Young Child Feeding (IYCF) practices which influence the nutritional status of children under two years.

Regarding complementary feeding, findings from the GDHS (2014) revealed that overall, only 15% of breastfed children were able to meet the minimum standards of feeding practices with respect to their dietary diversity score (DDS) and meal feeding frequency (MFF) [3]. Among non-breastfed children aged between 6 and 23 months, only 5% were fed with other milk or milk products at least twice a day, received the minimum meal frequency (MMF) of four solid or semi-solid foods and ate from at least four food groups, apart from the milk or milk products food group [3].

However, in Ghana, regional variations exist with respect to the proportions of children fed on a minimum acceptable diet (MAD)- ranging from 26.9% in the Central Region, 8.5% in the Upper East Region and 4.3% in the Eastern Region. In addition, there are rural–urban disparities with respect to all the IYCF indicators. Whereas 33.5% of children in urban settings were fed from four (4) or more food groups, only 23.6% met the minimum dietary diversity score (DDS) requirement. Similarly, 14.4% of urban children compared with 12.3% of rural children received minimum acceptable diet. Overall, only 13% of children aged between 6 and 23 months were fed a minimum acceptable diet [3]. The reasons behind the regional and habitat differences in MAD in Ghana can be mainly attributed to food insecurity issues confronting most households as a result of worse climatic conditions resulting in poor crop yield and harvest in these regions. In addition, the Eastern Region as a result of the mountainous nature of its geographical location and inaccessible roads makes it difficult to transport food stuffs to most of the districts within the region as compared with regions located in the coastal belt. These findings from the 2014 GDHS reveal that there exists a significant gap in explaining why, in spite of the implementation of the IYCF guidelines, policies and several interventions in Ghana, there is still low compliance with the recommended feeding practices.

Sub-optimal feeding and undernutrition in children is as a result of several complex, multifaceted and interconnected factors. Studies have revealed that factors at the community [6, 7], household [8, 9], maternal [8, 10] and individual [7, 8] levels influence the feeding practices and nutritional status of children. Given that a mother is a child’s primary caregiver and responsible for meal planning, cooking and feeding of children, a study that examines how maternal socio-economic characteristics influence the nutritional status of children under-two years is key towards addressing childhood undernutrition.

Furthermore, most of the previous studies that were conducted to assess the determinants of the nutritional status of children in Ghana focused on children under- five years [11–14] and these relationships are yet to be determined among children under two (2) years. Again, these previous studies did not focus on some maternal variables (parity, financial independence and participation in decision-making at the household level) and how they influence feeding practices and nutritional status of children under two (2) years. This study therefore focused on children under two years because it is an early stage in life to identify probable determinants of sub-optimal feeding practices and poor nutritional status before it results in irreversible outcomes of stunting and poor cognitive development. This is imperative because basic nutrition interventions when implemented early in life can avert a quarter of child mortalities and one-third of chronic malnutrition [15].

Therefore, this study assessed maternal factors associated with the various IYCF indicators (MDD and MAD) of infants and young children aged between 6 and 23 months.

In addition, the study assessed maternal factors that influence the nutritional status (using the anthropometric indicators of stunting, underweight and wasting) of infants and young children aged between 6 and 23 months.

## Methods

### Study design

The study adopted a cross-sectional design in which quantitative data were collected.

### Study setting

The Eastern Region of Ghana was selected for this study because, according to the GDHS (2015) report, only 4.3% of children residing in that region received a minimum acceptable diet compared to the national average of 13%. Specifically, this study was conducted in two districts − the Kwahu Afram Plains North District (KAPND) and the Kwahu Afram Plains South District (KAPSD) − which were ranked as the first and second districts with the highest prevalence of underweight children in the Eastern Region in both 2016 and 2017 (Eastern Region Health Directorate, 2016; 2017).

#### Study population

The study participants included 935 mothers with their under-two-year-old children who were randomly selected from 21 randomly selected Child Welfare Clinics (CWCs) in the two districts. These mothers accessed the CWCs for growth monitoring and promotion services for their young children.

#### Inclusion criteria

Eligibility criteria for infants included healthy singleton infants with ages between 0 and 23 months, gestational ages between 37 and 42 weeks, birthweight between 2,500 and 4,000 g, and without metabolic or physical problems. The gestational age and birth weight of the children were considered because available evidence indicates that a short gestational age, pre-term/premature or low birthweight infants are at higher risk of growth and nutritional deficits during the first year of life [16, 17]. The eligibility criteria for prospective study participants were assessed by checking the child’s health record booklet with the assistance of the nurses in-charge at the CWCs. Specifically, information on each child’s birthweight, birth defects or medication conditions was obtained from the health record booklet and formed the basis for determining their eligibility to participate in the study.

#### Exclusion criteria

Children who were twins were excluded from this study since the evidence regarding the differences between their nutritional status and that of singletons is inconsistent [18]. In addition, children whose twin siblings died during labour or birth were not considered. A child was also excluded if he/she had a chronic or congenital illness or any medical condition that interfered with either feeding (e.g. cleft palate) or the taking of body measurements.

#### Sample size

The sample size required for the study was estimated using the prevalence of underweight among children under-five years for the Kwahu Afram Plains North and South Districts in 2016, which are 22.0% and 16.3% respectively (District Health Directorate Report, 2016). The prevalence of underweight was used because it is a composite indicator reflecting both chronic and acute malnutrition and therefore, related to both stunting and wasting. This ensured that all the three indices of nutritional status were captured. On the average, prevalence for both the North and South districts was 19.2%, and the minimum sample size was estimated using the formula:

 $\mathrm n\:=\:\mathrm Z^{2\ast}\mathrm p^\ast(1-\mathrm p)/\mathrm e^2$ where Z = confidence level, *p* = proportion of underweight children in the combined districts and e = precision [19].

With a confidence level of 95%, Z = 1.96, e = 2.8% and *p* = 10.8%, the minimum sample size estimated was 765 children. Making a provision of 20% contingencies, a sample size of 912 (rounded up to 950) infants and children aged between 6 and 23 months was estimated for this study.

#### Sampling method

A two-stage sampling strategy was employed in this study. Firstly, a total number of 21 health facilities (constituting one-third of total health facilities) were randomly selected from a total of 63 health facilities within the two districts. Secondly, mothers and their children aged between 6 and 23 months were recruited from the 21 randomly selected health facilities for the study. The total number of infants and young children who visit the CWCs in the selected health facilities on a monthly basis was obtained. Based on this information, the study participants were randomly selected proportionally to obtain the sample size.

### Ethical considerations and participant approval

Ethical approval and clearance for the study was granted by the Dodowa Health Research Centre’s (DHRC) Institutional Review Board (IRB) of the Ghana Health Service (Reference/Identification: DHRCIRB/04/02/18) and the IRB of the University of Cape Coast (Reference/Identification: UCCIRB/CHLS/2018/02). Permission to conduct the study was also obtained from the Regional Health Directorate, the two District Health Management Teams (DHMT) and the Directors of Health Services in the 21 health facilities which were selected for the study. Informed consent to participate in the study was obtained from all study participants and mothers consented on behalf of their children. The participants consented to participant in the study by either thumb-printing or signing an informed-consent form. To ensure data confidentiality, participants were assigned unique identifiers instead of names.

All data collection methods were carried out in accordance with relevant ethical guidelines of the Ghana Health Service Ethical Review Committee and the University of Cape Coast Ethical Review Board. The regulations involved in the conduct of this research involving study participants also took into consideration the Helsinki Declaration.

### Study instrument

A structured questionnaire was used. The questionnaire solicited for information on socio-demographic characteristics of the mother such as marital status, parity, highest level of education attained, employment status and caregiver's autonomy (decision-making power and financial independence). In addition, information on background characteristics of the index child such as sex, age, birth order and birth weight were collected. Furthermore, information on each caregiver's feeding practices for the index child was obtained by administering a 24-h dietary recall and a seven-day food group frequency questionnaire (FGFQ).

### Anthropometric assessment of children

Anthropometric measurement was carried out to determine the nutritional status of the children by accessing for height-for-age z-score (HAZ), weight-for-age z-score (WAZ) and weight-for-height z-score (WHZ). The recumbent length of children who were unable to stand upright was measured using an infantometer (UNICEF model, item no. 0114500). The World Health Organization’s guidelines for taking weight and length/height in children were followed for all the anthropometric measurements [20]. Children’s heads were held vertically against the head plate with the back, body and legs straight and flat in the centre of the measuring board, the knees straightened, the heels and feet firmly positioned by the researcher vertically against the footplate. Both length and height measurements were taken to the nearest 0.1 cm. A beam balance scale was used to take their weights to the nearest 0.1 kg. All weighing scales were calibrated daily using certified standardized weight test loads of 10 kg and 25 kg by setting it to zero prior to taking weight measurements. It was ensured that all weights were measured with light clothing and no shoes. Both height and weight measurements were taken in duplicates and the average recorded.

### Dietary assessment of children

A single 24-h Dietary recall (24HDR) method and a seven-day food group frequency questionnaire (FFQ) were used to collect information on all foods (including school meals) and beverages consumed by the children. The mothers were asked to recall all the foods and drinks given to the child during the previous day and also the number of times that they fed their children the previous day. Information about the number of times (food group frequency) a child had eaten from a particular food group during the past seven days was collected using a food group frequency questionnaire. The seven food groups included grains/roots/tubers; legumes/nuts; dairy; flesh foods; eggs; vitamin A-rich fruits and vegetables; other fruits and vegetables [21]. Mothers were asked to recall the number of days in a week that children ate from each of the seven food groups.

Data collected using the 24HDR was used to estimate the minimum dietary diversity score (MDD) of the children. A child was considered to have met the requirement of being fed on a minimum dietary diversity if he/she ate from at least four out of the seven food groups during the day or night preceding the survey. The number of times that children had been offered meals in the past 24 h was used to estimate the minimum meal feeding frequency (MFF) of the children. The minimum acceptable diet (MAD) was estimated using the minimum DDS and the MFF. The children were sub-divided into four groups (less than 6 months, 6–8 months, 9–11 months, and 12 months and above) during the analysis of the feeding practices data to represent the variations in feeding recommendations for their different age groups. The scoring system for the IYCF indicators were dependent on the age group of each child and the current WHO feeding recommendations for that particular age group.

### Assessment of nutritional status

The WHO Anthro software (World Health Organization, 2011) was used to transform the weight and length/height of the infants into the growth indices -weight-for-age, weight-for-length and length-for-age z-scores. Infants with z-scores less than -2 standard deviations from the median reference length-for-age/ height-for-age, weight-for-height/weight-for-length and weight-for-age z-scores were classified as stunted, wasted and underweight, respectively.

### Assessment of child feeding indicators

#### Dietary diversity score (DDS)

The WHO recommends that children aged between 6 and 23 months should be given foods from at least four food groups each day to meet their minimum dietary diversity score. On the basis of this recommendation, a child in any age group who was not fed from any food group or fed from only three or less food groups in a day earned a score of 0. A score of 1 was assigned to a child who was fed from four food groups, and a score of 2 was given to children in the age groups 6–8 months and 9–12 months if more than 4 food groups are consumed. The scoring system that was applied in the study was based on the scoring pattern for the feeding indicator of dietary diversity score as applied in previous studies [22, 23]. A child aged between 13 and 23 months was given a score of 3 if she consumed more than four food groups within a 24-h period before the caregiver is interviewed. This is because of WHO’s emphasis on feeding children above 12 months from a wide variety of foods and gradually substituting their usual infant foods with family foods.

### Meal feeding frequency (MFF) score

Meal feeding frequency (MFF) score refers to the number of times that a child consumes solid or semi-solid foods within a day, including meals and snack. The MFF scoring pattern applied in this study was based on WHO’s recommendations on the number of meals that children belonging to the various age-groups should be fed daily in addition to breastfeeding and as described and applied in previous studies [22, 23]. On the basis of WHO’s recommendations, a score of zero (0) was given to children aged 6–8 months who were fed complementary foods 0–1 times, 1 when fed two times, and a score of 2 when fed more than two times (> 2) over a 24-h period prior to interviewing the mother. For children in the age groups of 9–12 months and 13–23 months, a score of 0 was awarded if a child was fed 0–2 times in a day, a score of 1 was given when a child ate complementary foods three times in a day, and a score of 2 was awarded when a child ate complementary foods more than three times in a day [21].

### Minimum acceptable diet (MAD)

It is a composite indicator for assessing feeding practices among 6–23 months old children and considers whether the child has been fed a minimum number of times (MFF) and on a diversified diet (MDD) daily [21]. Scoring and coding of the MAD indicator was done by applying the scoring pattern of previous studies [22, 23]

### Coding of maternal decision-making power and financial independence

A mother’s household decision-making autonomy was based on responses to 15 questions which covered themes on decisions in the respondent’s household about obtaining healthcare, large household purchases, visits to family or relatives, and child healthcare. Each of the questions had four (4) response options – respondent alone, respondent and husband/partner jointly, husband/partner alone and any other household member which were coded as 4, 3, 2 and 1 respectively. The total scores which were between 15 and 60 were re-categorised as low (15–30), medium/average (31- 45) and high (46–60) decision making power similar to the coding of mother’s household decision-making level in related studies [24, 25].

Control and access over finances or financial independence of a mother was assessed based on her responses to 12 questions. Seven (7) of the questions assessed a mother’s ability to have control over money to buy perishable food items, clothes, medicine, toiletries, jewelry, gifts for parents or other family members. Each of the questions had two response options namely- respondent has control (Yes) and no control (No). “Yes” and “No” were coded as 1 and 0 respectively. The other 5 questions assessed the mother’s ability to save a portion of the money she had earned, spend her earnings as she wished and have a say in how the household’s overall income should be spent. Each of these 5 questions were given three response options – no/never, yes/some of the time and yes/ all the time which were coded as 0, 1 and 2 respectively. The total scores ranged between 0 and 17 and were categorised into low (0–8), medium/average (9–12) and high (13–17) financial independence similar to the coding of levels in related studies [24, 26].

### Data quality control

The questionnaire was first developed in English and translated to the two local dialects (Ewe and Akan) and back to English to maintain its consistency and readjustments of inconsistent and inaccurate data. Prior to the actual data collection, the questionnaire was pretested on 20 women at Bruben CHPS Zone to ascertain the reliability, language clarity and simplicity of the tools. The questionnaire was checked for completeness daily by the supervisors and the principal investigator. The data collection period was between August and December, that is the minor rainy season, when food is usually in abundance to the beginning of the dry season (December) when food is a little bit scarce.

### Variables of the study

#### Dependent variables

Dietary diversity score (poor, average, good), minimum acceptable diet (adequate, inadequate) and anthropometric indicators (height-for-age z-score [not stunted, stunted], weight-for-age z-score [not underweight, underweight] and weight-for-height z-score [not wasted, wasted]).

#### Independent variables

Maternal- related variables which were categorical in nature included age (< 20, 20–30, 31–40, > 40) marital status (single [never married], cohabiting, married, separated/divorced, widowed) number of living children (parity) (1–3, 4–6, > 6), number of mothers with children under two years of age (1, 2, 3), employment status (not employed in the last 12 months, not currently employed but worked in past 12 months, currently employed),work status/schedule throughout the year (work throughout the year, work seasonally/part of the year, work only once in a while), form of remuneration/payment (cash only, cash and in kind, in kind only), mother’s highest level of education attained (none, primary, JHS/JSS, secondary, tertiary) decision making power (low, medium/average) and financial independence (low, medium/average, high).

### Statistical analysis

Socio-demographic characteristics of study participants were described using frequencies and their corresponding percentages. Multinomial logistic regression was used to examine the association between maternal-related factors and the outcome dietary diversity score (DDS) which was a categorical variable with three levels. Binary logistic regression was employed to determine the association between maternal-related factors and minimum acceptable diet (MAD) which was the outcome with two levels. Binary logistic regression was used to determine the influence of maternal factors on anthropometric indicators (height-for-age, weight-for-height and weight-for-age outcomes). The regression models were constructed by firstly determining the independent maternal factors that were significant in the bivariate models at the significance level of 0.05. Subsequently, all the maternal factors that were significant in the bivariate models were used to construct the multivariate models. All statistical tests were two-tailed and *p*-values < 0.05 were considered to be statistically significant. Stata software (version 15.0) was used for the statistical analysis.

## Results

### Socio-demographic characteristics of participants

Most of the mothers (65.6%) had number of children between 1 and 3 (Table 1). Approximately 75% (75.2%) and 36% (35.6%) were married and employed respectively. About 18% (17.9%) of the caregivers reported that they did not receive any remuneration for work they were engaged in at the time of the study. Most of the mothers (33.9%) had JHS/JSS and 17.8% of them had no formal education. Slightly above half of the mothers had average decision- making power (51.1%) and low financial independence (52.5%). With regard to the children, the proportion of females (50.2%) was slightly larger than that of the males (49.8%). The highest proportion of children were between the ages of 12 and 24 months (35.2%).

**Table 1:** Socio-demographic characteristics of mothers and children

Variable	Frequency	Percentage (%)
	***N* = 935**	
***Children’s characteristics***		
**Age (months), Mean (SD**)	9 (5.7)	
< 6	262	28.0
6 – 8	187	20.0
9 – 11	157	16.8
12 – 24	329	35.2
** Sex**		
Male	466	49.8
Female	469	50.2
***Mothers’ characteristics***		
**Maternal age (years), Mean (SD)**	28 (6.7)	
< 20 years	74	7.9
20–30 years	570	61.0
31 -40 years	243	26.0
> 40 years	48	5.1
** Marital status**		
Single (Never Married)	98	10.5
Cohabiting	83	8.8
Married	703	75.2
Separated/ Divorced	41	4.4
Widowed	10	1.1
** Number of Living Children (Parity)**		
1–3	614	65.6
4–6	282	30.2
> 6	39	4.2
** Number of mothers with children under two years of age (distributed by number of children cared for)**		
1	811	86.8
2	118	12.6
3	6	0.6
** Employment status**		
Not employed in the last 12 months before the survey	282	30.2
Not currently employed, but worked in past 12 months	320	34.2
Currently employed	333	35.6
** Work status/schedule throughout the year**		
Work throughout the year	322	34.4
Work seasonally/part of the year	313	33.5
Work only once in a while	300	32.1
** Form of remuneration/payment**		
Cash only	372	39.8
Cash and in Kind	302	32.3
In Kind only	94	10.0
Not paid	167	17.9
** Mother’s highest level of education attained**		
None	166	17.8
Primary	291	31.1
JHS/JSS	317	33.9
Secondary	92	9.8
Tertiary	69	7.4
** Decision making power**		
low	320	34.2
medium/average	479	51.2
high	136	14.6
** Financial independence**		
low	491	52.5
medium/average	352	37.7
high	92	9.8

*SD* Standard deviation

### Feeding practices of the children

The majority (63%) of the children had inadequate minimum dietary diversity score (63.0%) and highest proportion (77.9%) did not receive minimum acceptable diet (Table 2). However, most of them (59.6%) of them had adequate minimum meal frequency (Table 2).

**Table 2:** Information on child feeding indicators

Feeding Indicators	Frequency	Percentage (%)
	*N* = 935	
**Currently breastfeeding**		
Yes	848	90.7
No	87	90.3
**Dietary Diversity Score (DDS)**		
Poor	246	36.5
Average	178	26.5
Good	249	37.0
**Minimum Dietary Diversity Score (MDDS)**		
Adequate	249	37.0
Inadequate	424	63.0
**Food Group Frequency Score (FGFS) (Past 7 days)**		
Poor	172	25.6
Average	293	43.5
Good	208	30.9
**Meal Frequency (MF)**		
Poor	91	13.5
Average	181	26.9
Good	401	59.6
**Minimum Meal Frequency (MMF)**		
Adequate	401	59.6
Inadequate	272	40.4
**Minimum Acceptable Diet (MAD)**		
Adequate	149	22.1
Inadequate	324	77.9

In the multivariate multinomial logistic model, maternal factors that were associated with the DDS of children were marital status, employment status, work schedule throughout the year, mother’s highest level of education and financial independence of mothers (Table 3). Children whose mothers were married (AOR = 2.82, 95% CI: 1.45–5.52, *p* = 0.002) and those whose mothers were separated, divorced or widowed (AOR = 3.07, 95% CI: 1.11–8.48, *p* = 0.030) had increased likelihood of having a good DDS as compared to those whose mothers had never married. Regarding marital status, children of married mothers were 2.82 times more likely to have a good DDS compared with those whose mothers were single (AOR = 2.82, 95% CI: 1.45—5.52, *p* < 0.002). Similarly, children of mothers who were separated, divorced or widowed were 3.07 times more likely to have a good DDS compared with those whose mothers were single (AOR = 3.07, 95% CI: 1.11—8.48, *p* = 0.030) (Table 3). Children of mothers who were currently employed (AOR = 2.74, 95% CI: 1.61—4.68, *p* < 0.001) and those with mothers not currently employed but worked in the past 12 months (AOR = 1.68, 95% CI: 1.02—2.78, *p* = 0.042) had higher odds of having a good DDS. Children whose mothers worked only once a while were 48% less likely to have average DDS as compared to those whose mothers worked consistently throughout the year (AOR = 0.52, 95% CI: 0.28—0.98, *p* = 0.044). Children who had mothers with primary (AOR = 2.79, 95% CI: 1.55—5.02, *p* = 0.001), JHS/JSS (AOR = 2.65, 95% CI: 1.46—4.80, *p* = 0.001) and secondary or higher level of education (AOR = 3.15; 96% CI:1.57—6.33, *p* = 0.001) were more likely to have a good DDS as compared to those whose mothers had no formal education. In addition, children whose mothers had secondary or higher level of education (AOR = 2.02; 96% CI:1.00—4.06, *p* = 0.049) had increased likelihood of having an average DSS. Children of mothers with average (AOR = 1.64, 95% CI: 1.05—2.55, *p* = 0.029) and high (AOR = 1.55, 95% CI: 1.01—2.38, *p* = 0.045) financial independence statuses were more likely to have a good DDS compared to those of mothers with a low level of financial independence (Table 3).

**Table 3:** Multivariate multinomial logistic regression for the association between maternal-related factors and dietary diversity score (DDS)

	**Average DDS vs. Poor DDS**		**Good DDS vs. Poor DDS**	
**Variable**	**AOR (95% CI)**	***p*-value**	**AOR (95% CI)**	***p*-value**
**Marital status**^§^				
Single (Never Married)	1 (Reference)		1 (Reference)	
Cohabiting	1.48 (0.63—3.45)	0.367	1.47 (0.58—3.70)	0.413
Married	1.37 (0.70—2.66)	0.358	2.82 (1.45—5.52)	0.002
Separated/ Divorced/widowed	1.19 (0.40—3.57)	0.751	3.07 (1.11—8.48)	0.030
**Number of Living Children** **(Parity)**				
1–3	1 (Reference)		1 (Reference)	
4–6	1.12 (0.72—1.77)	0.610	1.14 (0.74—1.74)	0.557
> 6	0.31 (0.08—1.13)	0.075	0.55 (0.20—1.52)	0.252
**Mother’s Employment status**^§^				
Not employed in last 12 months before the survey	1 (Reference)		1 (Reference)	
Not currently employed, but worked in past 12 months	1.41 (0.85—2.34)	0.185	1.68 (1.02—2.78)	0.042
Currently employed	1.17 (0.67—2.03)	0.586	2.74 (1.61—4.68)	< 0.001
**Work status/schedule throughout the year**^§^				
Work throughout the year	1 (Reference)		1 (Reference)	
Work seasonally/part of the year	0.88 (0.52—1.49)	0.644	0.87 (0.52—1.44)	0.588
Work only once in a while	0.52 (0.28—0.98)	0.044	1.07 (0.59—1.96)	0.816
**Mother’s highest level of education attained**^§^				
None	1 (Reference)		1 (Reference)	
Primary	1.13 (0.63—2.05)	0.678	2.79 (1.55—5.02)	0.001
JHS/JSS	1.61 (0.90—2.88)	0.105	2.65 (1.46—4.80)	0.001
Secondary +	2.02 (1.00—4.06)	0.049	3.15 (1.57—6.33)	0.001
**Decision making power**				
Lowest	1 (Reference)		1 (Reference)	
Middle	0.93 (0.56—1.52)	0.759	1.29 (0.81—2.08)	0.285
Highest	1.44 (0.71—2.95)	0.316	1.99 (1.00—3.97)	0.051
**Financial independence**^§^				
low	1 (Reference)		1 (Reference)	
average	1.64 (1.05—2.55)	0.029	1.55 (1.01—2.38)	0.045
high	1.07 (0.47—2.48)	0.867	1.79 (0.84—3.79)	0.131

^§^ Factors with overall *p*-value < 0.05; Secondary +  = secondary education or higher

In the multivariate logistic regression, the maternal factors that were associated with the outcome MAD were employment status, highest level of education attained and decision-making power (Table 4). The children of mothers who were currently employed (AOR = 3.07, 95% CI: 1.71—5.49, *p* < 0.001) and those whose mothers were not employed during the survey but worked in the past 12 months prior to the survey (AOR = 1.96, 95% CI: 1.10—3.47, *p* = 0.022) had increased likelihood of receiving MAD as compared to those whose mothers were not employed in the last 12 months prior to the survey. Children whose mothers had primary (AOR = 2.44, 95% CI = 1.24—4.75, *p* = 0.009), JHS/JSS (AOR = 2.24, 95% CI = 1.15—4.33, *p* = 0.017) and secondary or higher level of education (AOR = 2.86, 95% CI = 1.42—5.78, *p* = 0.003) were more likely to receive MAD compared to children whose mothers had no formal education. Additionally, children of mothers with an average decision-making autonomy had higher odds of receiving MAD (AOR = 1.68, 95% CI = 1.02—2.76, *p* = 0.040) (Table 4).

**Table 4:** Logistic regression model for the association between maternal-related factors and minimum acceptable diet (MAD)

	**Multivariate regression**	
**Variable**	**AOR (95% CI)**	***p*-value**
**Maternal age (years)**		
< 20	1(Reference)	
20–30	2.26 (0.65—7.82)	0.200
Above 30	3.27 (0.92—11.58)	0.066
**Employment status**^§^		
Not employed in last 12 months before the survey	1 (Reference)	
Not currently employed, but worked in past 12 months	1.96 (1.10—3.47)	0.022
Currently employed	3.07 (1.71—5.49)	< 0.001
**Work status/schedule throughout the year**		
Work throughout the year	1 (Reference)	
Work seasonally/part of the year	1.06 (0.67—1.68)	0.815
Work only once in a while	1.06 (0.58—1.91)	0.854
**Highest level of education attained**^§^		
None	1 (Reference)	
Primary	2.44 (1.24—4.75)	0.009
JHS/JSS	2.24 (1.15—4.33)	0.017
Secondary +	2.86 (1.42—5.78)	0.003
**Decision-making power**^§^		
Lowest	1 (Reference)	
Average	1.68 (1.02—2.76)	0.040
Highest	1.34 (0.73—2.57)	0.328

§ Factors with overall *p*-value < 0.05 in the multivariate analysis

Marital status, number of living children (parity), number of children under 2 years of age, form of remuneration/payment, mother’s nutritional status (BMI), Financial independence and mother’s nutritional knowledge - are not shown in Table 4

### Association between maternal-related factors and children’s nutritional status

The various multivariate binary logistic regressions in Table 5 shows that the number of children under two years of age that a mother had was a predictor of stunting (HAZ) and underweight (WAZ). Children of mothers who had 2 or 3 children under two years were more likely to be stunted (AOR = 1.88, 95% CI: 1.23—2.87, *p* = 0.003) in comparison with those whose mothers had only one child under two years of age. Similarly, children whose mothers had 2 or 3 children under 2 years had higher odds of being underweight (AOR = 1.53, 95% CI: 1.02 – 2.30, *p* = 0.039). Children of currently employed mothers were 29% less likely to be stunted compared to those whose mothers were not employed in the last 12 months prior to the survey (AOR = 0.71, 95% CI: 0.47 – 0.97, *p* = 0.043). The children whose mothers had primary (AOR = 0.55, 95% CI: 0.35 – 0.88, *p* = 0.012), JHS/JSS (AOR = 0.55, 95% CI: 0.36 – 0.89, *p* = 0.015) and secondary or higher level (AOR = 0.49, 95% CI: 0.28 – 0.86, *p* = 0.014) of education had lower odds of being wasted as compared to those whose mothers had no formal education. Additionally, children of mothers who had secondary or higher level of education had reduced risk of being underweight (AOR = 0.59, 95% CI: 0.36 – 0.97, *p* = 0.040). Compared with children whose mothers had low financial independence, those whose mothers had average financial independence were 44% less likely to be wasted (AOR = 0.66, 95% CI: 0.45 – 0.95, *p* = 0.025).

**Table 5:** Logistic regression for the association between maternal-related factors and anthropometric indicators

	**Multivariate regression (HAZ)**		**Multivariate regression (WHZ)**		**Multivariate regression (WAZ)**	
**Variable**	**AOR (95% CI)**	***p*-value**	**AOR (95% CI)**	***p*-value**	**AOR (95% CI)**	***p*-value**
**Maternal age (years)**						
< 20						
20–30						
Above 30						
**Marital status**						
Single ( Never Married)						
Cohabiting						
Married						
Separated/ Divorced/widowed						
**Number of Living Children (Parity***)*						
1–3			1(reference)		1(reference)	
4–6			1.30 (0.91—1.85)	0.155	1.25 (0.91—1.71)	0.162
> 6			1.75 (0.84—3.65)	0.138	1.86 (0.94—3.69)	0.046
**Number of children under two years of age**^a^						
1	1 (reference)				1 (reference)	
2/ 3	1.88 (1.23–2.87)	0.003			1.53 (1.02—2.30)	0.039
**Employment status**^§^						
Not employed in last 12 months before the survey	1 (reference)					
Not currently employed, but worked in past 12 months	0.93 (0.63- 1.36)	0.701				
Currently employed	0.71(0.47- 1.07)	0.043				
**Work status/schedule throughout the year**						
Work throughout the year					1 (reference)	
Work seasonally/part of the year					0.95 (0.66—1.37)	0778
Work only once in a while					1.39 (0.97—1.97)	0.071
**Form of remuneration/payment**						
Cash only						
Cash and in Kind						
In Kind only						
Not paid						
**Mother’s highest level of education attained**						
None			1 (reference)		1(reference)	
Primary			0.55 (0.35—0.88)	0.012	0.79 (0.52—1.19)	0.260
JHS/JSS			0.56 (0.36 -0.89)	0.015	0.74 (0.49—1.11)	0.146
Secondary +			0.49 (0.28 -0.86)	0.014	0.59 (0.36 -0.97)	0.040
**Mother's nutritional Status (BMI)**						
Under Weight						
Normal weight						
Overweight						
Obese/ very obese						
**Decision making power**						
Lowest						
Middle						
High						
**Financial independence**^a^						
Low	1 (reference)		1 (reference)			
Middle	0.89 (0.64–1.26)		0.66 (0.45–0.95)	0.025		
High	0.58 (0.30–1.11)		1.14 (0.70.-1.86)	0.606		
**Mother’s nutritional knowledge**						
Low/medium (0–15)						
High (16–23)						

^a^ Factors with overall *p*-value < 0.05 in the multivariate analysis; HAZ, WHZ and WAZ represents stunting, wasting and underweight in children respectively

## Discussion

The findings indicate that 63.0% of the children had an inadequate minimum dietary diversity score (DDS), implying that the diet of a lot of the children in the two districts lack diversity. The finding is similar to a study conducted in Ghana in which only 24.7% of the children had a dietary diversity score of at least 4 out of 7 food groups [11]. However, unlike the situation with respect to the DDS, more than 50% of the children met the minimum feeding frequency (MFF) requirement recommended for their age. In other related studies, it was also observed that whereas most children met the requirement for the minimum feeding frequency, less than half were fed on meals prepared from a wide range of food groups [27, 28]. This could be because although most of the children may be eating meals frequently in the day, these meals are monotonous cereal and starch-based diets, without much variety. Another possible reason that can be adduced for the high MFF but low DDS is that, in the current population, most of the households mainly feed on their farm produce. If they do not grow different food crops, there is a high probability that they would be feeding on monotonous meals. It was observed in a study conducted in rural Ethiopia that households which do not feed mainly on their own agricultural produce are more likely to consume more diverse diets [29]. Likewise, another study revealed that dietary diversity score was significantly higher among non-farming households than farming households [30]. The implication of this finding is the need to educate farming households in rural settings on the importance of feeding children on diversified foods and not only from farm produce or advocate for interventions that encourage diversification of farm production.

A related finding was that most of the children (77.9%) were not receiving a minimum acceptable diet (MAD), largely because the majority of the children were not being fed on highly diversified meals from different food groups although the data suggested that they ate frequently during the day. These results confirm previous studies that most children with ages between 6 and 23 months do not receive a minimum acceptable diet, mainly because they are not fed from a wide range of different food groups, in spite of the majority meeting the requirement for a minimum meal frequency [27, 31].

The study revealed that children whose mothers have highest level of education and also being employed throughout the year are received an adequate DDS and MAD. This finding corroborates the findings of studies conducted in the Philippines [32], Timor-Leste [33], six south Asian countries [34], Ethiopia [35] and Kenya [36]. This finding could be attributed to the ability of educated mothers to read, understand and apply information from nutrition education materials provided when they access child health facilities. Besides, women who attain high levels of education may be more likely to have access to more nutritional information and understand educational messages delivered through different media outlets. This ultimately assist them to follow optimal child feeding practices. In addition, highly educated mothers are more likely to secure jobs with regular monthly salaries which empower them to have more resources to provide their children with adequate and varied meals. It is therefore not surprising that this study observed a relationship between the educational level of a mother and her employment status. A meta-analysis of 50 Demographic and Health Surveys, reported that children of employed women, compared with those of unemployed women, had higher odds of achieving a minimum DDS and MAD [37]. However, the findings are contrary to conventional wisdom advocating for maternal employment in other studies in Bangladesh [38] and Vietnam [39], where maternal employment was found to be detrimental to child’s feeding and nutritional status. This observation was made because although maternal employment increased the income levels of mothers, it ended up decreasing the quantity of time that was spent caring for the nutritional needs of their children.

Children of married mothers were found to be more likely to have a good DDS and be fed on a minimum acceptable diet, compared with those whose mothers were single. These findings are in line with that of studies conducted in India [40], Ethiopia [41] and Benin [42] in which children of single mothers had low DDS and MFF. This finding accentuates the important role of fathers or husbands in ensuring that their children are optimally fed, and the entire household is food-secured. Another explanation is the high poverty levels among single mothers who often single-handedly have to provide for all the needs of their children, including their dietary intakes. This finding is contrary to observations in studies conducted in Ethiopia [43] and China [44] that children of single mothers have a better DDS compared with those of married mothers. The differences in these findings could be attributed to the time spent by mothers to care for male-partners, sometimes to the disadvantage of children, which may adversely influence their nutritional intakes. In other cases, married women, particularly in impoverished settings*,* who are likely to be confronted by food insecurity may aim at pleasing their husbands by feeding them first, giving them the best part of the meal and large portion sizes, to the detriment of their children and themselves. This assertion is confirmed by an earlier study which found that, mothers in poverty-stricken areas in Bangladesh feed their husbands first and later on eat the leftovers with their children [45].

One of the findings also revealed that children of mothers with at least an average financial independence were more likely to have a good DDS, just as observed in similar studies in Lao People's Democratic Republic [24] and Uganda [25]. This might be explained by the fact that such mothers were employed and therefore in a better position to provide nutritious and highly diversified diets for their children. Indeed, the data showed that more than 65% of the mothers with high financial independence were employed at the time of the study, whereas majority (70.7%) of the mothers with low financial independence were not employed. In situations where women are uneducated and unemployed, they are likely to depend on their spouses for financial resources to meet their own needs and those of their children. Such mothers may lack the purchasing power to provide a sufficiently varied diet for their children. In addition, their opinions on financial decisions and the allocation of resources in the household may not be considered by the head of the household, mainly because they may hardly contribute any monetary resources to the total income of the household and its upkeep. Again, such women with low autonomy may not even have much control over their own money earned from selling farm produce or from their small businesses.

Similarly, mothers who had at least an average ability to participate in decision-making at the household level were more likely to feed their children on a minimum acceptable diet as reported in related studies conducted in Nepal [46], Timor-Leste [33] and Bangladesh [47]. A possible explanation for this finding is that when mothers are involved in household decision-making, it could empower them to feed diversified foods to ensure optimal growth of their children. Another explanation is that in this study population, majority of mothers (78.0%) reported that their husbands made final decisions on how the household income should be used. Therefore, assuming a low proportion of the household income is allocated to food then the one in charge of preparing food, most probably the mother, may have to ensure that the money she had at hand met their dietary needs.

With respect to the association observed between maternal factors and the anthropometric indicators, parity of a mother was a significant predictor variable of underweight and stunting in children, corroborating previous studies [48, 49]. This could be due to the fact that, as the number of children increases, it causes a strain on family resources, as well as predisposing the children more to growth failures. A possible reason is that having a large number of children may lead to competition for both household resources and mother’s time and strength required for care-giving. Competition for the limited available dietary resources at home might cause each child to receive sub-optimal care, which includes feeding frequently and adequately on different nutritious food sources. In the present study, the highest level of educational attainment by a mother was a significant predictor variable of both underweight and wasting in children. This finding confirms the results of previous studies that a high educational level of mothers impacts the nutritional status of children [50–53]. There are plausible explanations as to why a higher maternal education reduces the risk of wasting and underweight among children. First, mothers who are educated to a higher level are at an advantage in processing interpreting and applying nutrition and health information that concerns their children's nutritional and health needs. Such information as is likely to be possessed by educated mothers can enable them to make informed decisions about their children’s nutrition and health care, thus leading to improved child and better health outcomes, such as a reduced risk of wasting and underweight in children. The study revealed that mother's current employment status and financial independence have influence on stunting, and this finding is in line with findings from other developing countries, such as the Lao People's Democratic Republic [24], Malawi [54] and India [55]. The plausible explanation might be that employed mothers may earn a regular income and use it beneficially for their children's health and nutrition.

In the current study, a significant association was found between a mother’s employment status and her financial independence. Further analysis of the data revealed that more than 65% of the mothers with high financial independence were employed at the time of the study whereas majority (70.7%) of the mothers with low financial independence were not employed at the time of the study. These findings suggest that the financial status of mothers who are employed improves and ultimately have a positive impact on their children’s dietary intakes and growth. However, studies carried out in Tanzania [56], Nepal [57] and Bangladesh [38] have found out that unemployed mothers are less likely to have stunted children, compared with employed mothers. The possible reason attributed to this finding by some of the authors is that unemployed mothers may have adequate time to provide their children with nutritious meals. Hence, this could have contributed to the prevention of stunting.

In addition, findings from the current study indicate that a mother's ability to actively participate in decision making at the household level protects her children from wasting. Findings of studies conducted in Nigeria [58], (Bangladesh, Nepal, Pakistan, and Myanmar) [59] and Burkina Faso [60] supports this finding. The possible reason attributed to this finding is that mothers with high decision-making power have control over resources and are likely to take decisions independently. This may subsequently influence choices positively to promote the survival and growth of their children. The findings thus contribute data for rural farming settings which can guide development of nutrition interventions for mothers to help tackle the problems of childhood undernutrition. For example, the findings suggest that educating and empowering mothers to be gainfully employed in order for them to be financially independent is vital in improving their children’s nutritional status.

One of the strength of this study is that, the sex distribution of children who participated in this study reflects the national situation of having slightly more females than males. Additionally, two height and weight measurements were taken on each child to minimize measurement variability and great emphasis was placed on daily calibration of the weight measuring instruments. Maternal autonomy factors like financial-independence and decision-making power were included in this study unlike previous related studies in Ghana. Although, strengths of this study have been outlined, it also has some few limitations. The study has a limitation of being cross-sectional and therefore causality cannot be established. Besides, the use of the 24-h dietary recall tool and a seven-day food group frequency questionnaire (FFQ) to assess dietary intakes relied on participants’ self-reported data, which were prone to recall and social desirability biases.

Since this is a cross-sectional study, it will be appropriate to conduct further studies which have longitudinal designs. Such longitudinal studies will enable researchers to assess for seasonal variations and its impact on feeding and nutritional status of children. Again, in future studies, a form for recording daily dietary intake of children could be given to individuals in selected households who can read and write to assist mothers to complete, (especially in cases where mothers are unable to read or write). By employing such a method, recall bias of children’s dietary intake is likely to be reduced among mothers. Also, another suggestion for further studies is to assess the influence of other maternal factors such as nutritional knowledge, access to nutrition education materials, counselling services and maternal dietary diversity scores on dietary intake and nutritional status of children. Future studies could assess how the other dimensions of maternal autonomy such as freedom of association, freedom of physical movement, access to economic resources/control over assets, barriers to health care access and attitude toward violence (wife-beating by husband, and experience of violence at home) influence the nutrition of children.

## Conclusions

Maternal factors such as a high educational level, stable employment, decision-making power at the household-level, financial independence and low parity contribute to ensuring optimal dietary intakes and improved nutritional status of young children. Therefore, education on the importance of child nutrition, family planning, crop diversification, women empowerment in decision-making, providing employment opportunities for mothers and improvement in girl-child education are recommended in child nutrition health interventions in rural settings.

## Data Availability

The datasets generated and/or analyzed during the current study are not publicly available due to some sensitive information on women autonomy issues in the cultural setting of participants which could compromise the privacy and anonymity of mothers who participated in the study but are available from the corresponding author (cbuxton@ucc.edu.gh) or the University of Cape Coast, Institutional Review Board at irb@ucc.edu.gh on reasonable request.

## Abbreviations

24HDR: 24-Hour Dietary recall
AOR: Adjusted Odds Ratio
CWC: Child Welfare Clinics
DHMT: District Health Management Teams
FFQ: Food frequency questionnaire
GDHS: Ghana Demographic Health Survey
GHS: Ghana Health Service
GSS: Ghana Statistical Service
HAZ: Height-for-age
IYCF: Infant and Young Child Feeding
KAPND: Kwahu Afram Plains North District
KAPSD: Kwahu Afram Plains South District
LAZ: Length-for-age
MAD: Minimum Acceptable Diet
MDD: Minimum Dietary Diversity
MFF: Minimum Feeding Frequency
MMF: Minimum Meal Frequency
UNICEF: The United Nations Children’s Fund
WAZ: Weight-for-age z-score
WHO: World Health Organization
WHZ: Weight-for-height z-score
WLZ: Weight-for-length z-score
